# From Variant to Biomarker in NSCLC Immunotherapy Resistance: Multiomics Evidence Chains and Accountable AI Integration

**DOI:** 10.1155/humu/6434376

**Published:** 2026-05-21

**Authors:** Yiqing Jiang, Na Wang, Qin Zeng, Jun Liu, Xiaoqin Liu, Guili Cao

**Affiliations:** ^1^ Department of Oncology, First People′s Hospital of Zigong, Zigong Medical Science Academy, Zigong, China

**Keywords:** artificial intelligence, genetic variation, immunotherapy resistance, multiomics, non–small cell lung cancer, PD-L1, tumor mutational burden

## Abstract

Immune checkpoint inhibitors have become integral to the management of non–small cell lung cancer (NSCLC), yet both primary and acquired resistance remain frequent and are only partially captured by routine biomarkers such as programmed death‐ligand 1 (PD‐L1) immunohistochemistry and tumor mutational burden (TMB). Resistance is increasingly viewed as a multiaxis functional phenotype shaped by antigenicity and neoantigen quality, antigen processing and presentation competence, interferon signaling and adaptive resistance programs, tumor–immune spatial organization, suppressive myeloid/stromal ecosystems, and metabolic constraints that limit effector function. Multiomics profiling provides a practical route to translate genomic event anchors into reproducible, mechanistically interpretable biomarker outputs by assembling coherent evidence chains across genomics, transcriptomics, epigenomics, proteomics, and metabolomics, complemented by spatial assays, digital pathology, and imaging‐derived surrogates.

## 1. Introduction

### 1.1. Clinical Expansion of Immune Checkpoint Blockade and Persistent Heterogeneity

Immune checkpoint blockade has expanded across non–small cell lung cancer (NSCLC) stages and lines of therapy, whereas substantial heterogeneity in clinical benefit has persisted [1, 2]. Durable responses have been achieved in a subset of patients, whereas primary resistance and early progression remain frequent [3, 4]. Acquired resistance has also been observed after initial benefit, highlighting dynamic tumor–immune coevolution under therapeutic pressure.

The dominant biomarkers in current practice—programmed death‐ligand 1 (PD‐L1) immunohistochemistry and tumor mutational burden (TMB)—have supported decision‐making in specific contexts but have demonstrated variable performance across cohorts, regimens, and assay platforms [5–7]. PD‐L1 expression is inducible and spatially heterogeneous, with measurement influenced by sampling site, timing, intratumoral variability, and antibody clone [8]. TMB is assay dependent and is affected by panel size, coverage, sequencing depth, artifact filtering, germline subtraction, and calibration strategy [9]. Importantly, neither marker captures several determinants that have repeatedly been implicated in NSCLC immunotherapy response, including the integrity of antigen processing and presentation, competence of interferon signaling, spatial accessibility of tumor nests, the organization of suppressive myeloid/stromal ecosystems, and metabolic constraints within the tumor microenvironment [10]. The recurring discordance between PD‐L1/TMB and clinical outcomes has reinforced the view that resistance is not governed by a single molecular dimension. A shift toward mechanistically anchored functional phenotypes has therefore been encouraged. Multiomics profiling is well suited to this objective, not because additional layers automatically confer robustness, but because coherence across layers can be used to distinguish stable biology from cohort‐specific correlation and technical artifacts [11, 12]. In this framework, genetic variation serves as an event anchor for mechanistic hypotheses, whereas transcriptomic, epigenomic, proteomic, and metabolomic layers jointly characterize immune programs, regulatory plasticity, execution‐level states (including antigen presentation and signaling), and microenvironmental constraints that impair effector function [13, 14]. Spatial and imaging modalities—such as single‐cell and spatial transcriptomics, multiplex immunohistochemistry, digital pathology (pathomics), and radiomics—complement these data by resolving tissue architecture and lesion‐level heterogeneity [15]. Genotype‐defined clinical subgroups add further complexity. Driver alterations (e.g., EGFR activating mutations, ALK fusions, ROS1 fusions, MET exon 14 skipping, and RET fusions) and common oncogenic contexts (e.g., KRAS mutations) have been associated with distinct immune ecosystems in retrospective analyses and clinical practice [16–19]. Coalterations involving STK11, KEAP1, TP53, PTEN, and others have been linked to differences in inflammatory signaling and checkpoint response [20–23]. However, deterministic stratification by genotype alone has been undermined by within‐context heterogeneity, lesion‐level diversity, and therapy‐induced evolution. A more transportable approach is therefore to use genomic context as a prior, whereby functional immune states are measured and validated with multiomics evidence.

**Figure 1:**
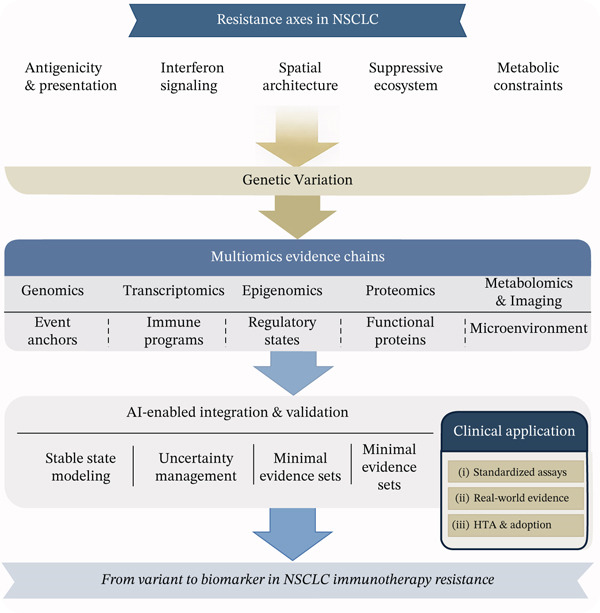
From variant to biomarker in NSCLC immunotherapy resistance: Multiomics evidence chains and accountable AI integration.

Figure 1 summarizes the proposed multiaxis resistance framework and the multiomics evidence chain from genetic variants to deployable biomarker outputs, together with an accountable AI integration strategy.

## 2. Immunotherapy Resistance as a Multiaxis Functional Phenotype

For biomarker development, resistance is more informative when framed as a set of measurable biological axes rather than reduced to a binary outcome, because nonresponse in NSCLC commonly arises from multiple, co‐occurring defects [24].

Antigenicity is one major axis of resistance. Although neoantigen burden shapes immune recognizability, TMB alone does not adequately capture antigen quality or effective presentation and is therefore an incomplete surrogate [25]. Effective antigenic exposure depends on whether neoantigens are clonal versus subclonal, whether mutant peptides are efficiently processed and presented, and how consistently they are shared across lesions [26]. Consequently, tumors with high TMB dominated by subclonal mutations may display fewer actionable clonal neoantigens than lower TMB tumors with early truncal events, and incorporating calibrated mutational‐process features (e.g., smoking‐ or APOBEC‐associated patterns and DNA repair deficiency signals) can provide a more faithful representation of the antigenic landscape than a single scalar metric [27–29]. Antigen processing and presentation competence determines immune visibility. Integrity of MHC Class I pathway components, including HLA Class I expression and assembly machinery, *β*2‐microglobulin, antigen processing transporters, and peptide loading complexes, is required for CD8 T‐cell recognition [30–33]. Genetic disruption, allele‐level alterations, or regulatory silencing of presentation components can decouple mutation‐derived antigenicity from immune visibility and may explain nonresponse in antigen‐rich tumors [34]. Interferon signaling competence shapes response by supporting immune activation and antigen presentation, yet it can also trigger adaptive resistance programs, including PD‐L1 upregulation and additional negative regulatory circuits [35]. Resistance may reflect either loss of interferon responsiveness or persistence of adaptive inhibitory programs that suppress effector function within an inflamed tumor. Consequently, discriminating between “inflamed and responsive” and “inflamed but adaptively resistant” is essential and cannot be inferred from PD‐L1 expression alone.

Tumor–immune spatial architecture critically shapes immune access. The mere presence of effector cells does not ensure their proximity to malignant nests; instead, immune cells may be sequestered in the peritumoral stroma while tumor nests remain inaccessible, consistent with “immune exclusion” [36, 37]. Stromal organization, dense extracellular matrix, fibroblast‐driven programs, and TGF‐*β*–associated signaling can establish physical and signaling barriers that prevent immune cells from accessing tumor nests, explaining why bulk assays may indicate inflammation despite persistent resistance [38]. Suppressive myeloid and stromal ecosystems can render effector T cells functionally ineffective despite their presence within tumors. Myeloid populations and stromal programs mediate suppression through cytokine and chemokine signaling, expression of inhibitory checkpoint ligands, sequestration of tumor antigens, and metabolic competition that constrains effector cell function [39]. These ecosystems can dominate resistance phenotypes and contribute to infiltrated yet ineffective immune microenvironments. Cytotoxic activity and proliferative capacity can be compromised by metabolic constraints such as hypoxia, lactate‐associated acidification, amino acid depletion, and lipid remodeling, while suppressive differentiation is concurrently favored [40]. In particular, lactate accumulation and extracellular acidification may impair T‐cell effector function and cytokine production, lipid metabolic rewiring may support tumor adaptation while promoting dysfunctional or suppressive immune states, and tryptophan catabolism through the kynurenine pathway may further limit effective antitumor immunity by reinforcing immunoregulatory programs [41–43].

When represented in combination, these axes enable the derivation of functional phenotypes that more directly approximate resistance mechanisms than any single biomarker. The objective of multiomics biomarker development is therefore to construct compact, coherent measurement sets that jointly capture these axes and support transportable performance under external validation.

## 3. Multiomics Evidence Chains From Genetic Variation to Biomarker Outputs

Multiomics biomarker development is most effective when each layer is assigned a specific role within an evidence chain. Genomics provides the input for mechanistic priors by identifying recurrent alterations linked to antigenicity, immune escape, and signaling competence. Transcriptomic and epigenomic data then indicate whether these priors are expressed as immune‐state programs or regulatory plasticity. Proteomic readouts test whether these programs are executed at the level of antigen presentation and signaling activity. Metabolomic readouts further determine whether the tumor microenvironment imposes constraints such as hypoxia, acidification, nutrient depletion, or lipid remodeling that suppress effective antitumor immunity. The resulting output is a cross‐layer, mechanistically interpretable biomarker framework that identifies the dominant resistance axis rather than relying on a single surrogate marker. This framework is particularly relevant in NSCLC immunotherapy because PD‐L1 and TMB may be insufficient when resistance is driven by processes they do not capture, such as impaired antigen presentation, limited immune access to tumor nests, dominant myeloid or stromal suppression, or microenvironmental metabolic restriction [44–46]. At the genomic level, recurrent and measurable genetic events provide an interpretable basis for formulating mechanistic hypotheses related to antigenicity, immune escape, and signaling competence. TMB provides only a coarse approximation of antigenicity and requires explicit calibration, as absolute values and decision thresholds vary with assay design and bioinformatic pipelines. Mutational signatures—such as smoking‐related patterns or APOBEC activity—may add mechanistic context by informing how the neoantigen repertoire was generated; however, these features should be incorporated only when they are reproducible across analytical workflows and show incremental benefit in independent external cohorts [47]. Importantly, antigenicity alone is insufficient. Inferences drawn from mutation‐based proxies are most convincing when complemented by “immune competence” checks—specifically, evidence that antigen processing and presentation, as well as interferon responsiveness, remain intact—ideally corroborated by downstream expression or protein‐level readouts [48]. Genotype‐defined contexts (e.g., EGFR/ALK, KRAS and common coalterations such as STK11, KEAP1, and TP53) are therefore best treated as priors that guide which resistance axes should be interrogated, rather than deterministic classifiers of immunotherapy benefit. Genomic interpretation is further complicated by clonality and spatial heterogeneity [49]. When feasible, multiregion sampling or longitudinal profiling may strengthen inference; when such data are unavailable, clonality‐aware summaries and cautious interpretation are warranted to avoid overextrapolation from a single biopsy [50].

Transcriptomics then provides a direct readout of immune‐state programs but must be interpreted with attention to cellular composition in bulk assays. Compact, interpretable modules—capturing interferon signaling, cytotoxic effector activity, antigen presentation expression, stromal/TGF‐*β*–linked exclusion, myeloid‐associated suppression, and tumor‐intrinsic stress or metabolic programs—tend to be more transportable than long gene lists, particularly when purity adjustment and conservative use of deconvolution are applied [51, 52]. Single‐cell and spatial transcriptomics are especially valuable during development to assign signatures to their cellular sources and to distill them into minimal marker sets that can be deployed in bulk or targeted assays, whereas spatial methods can directly validate exclusion and niche organization [53]. Epigenomic profiling can further clarify resistance states that arise without obvious new coding alterations by capturing regulatory competence and plasticity. DNA methylation and chromatin accessibility can indicate whether antigen presentation and interferon programs are epigenetically constrained or poised for activation, and these signals are most persuasive when they align with transcriptomic programs and, where feasible, proteomic confirmation [54, 55]. Proteomics and digital pathology provide execution‐level validation—beyond PD‐L1 alone—through assessment of presentation components, interferon pathway effectors, and inhibitory or suppressive signaling, and they offer a practical path to standardized panels [56, 57]. Metabolomics adds functional evidence for microenvironmental constraints such as lactate‐associated acidification, tryptophan–kynurenine pathway activity, hypoxia‐linked rewiring, and lipid remodeling, although careful preanalytical control is required given sensitivity to sampling and systemic confounders [58–61]. Imaging‐derived radiomics can contribute complementary, noninvasive context on lesion heterogeneity and hypoxia/necrosis–related patterns, but it is best viewed as supportive rather than a substitute for molecular profiling [62]. Ultimately, the objective is not to maximize dimensionality, but to derive a parsimonious, interpretable evidence set that remains reliable under realistic patterns of missing data. In practice, such sets integrate calibrated antigenicity and mutational‐process features, core checks of immune competence (including antigen processing/presentation and interferon responsiveness), compact state modules that distinguish immune activation from exclusion or suppression, and a limited number of execution‐ or constraint‐level confirmations. Within this framework, biomarker readouts are most defensible when reported as structured resistance phenotypes rather than as single‐scalar expectations based solely on PD‐L1 or TMB.

## 4. Applying Multiomics to Recurrent NSCLC Immunotherapy Patterns

To make multiomics biomarkers more useful in practice, recurring NSCLC immunotherapy scenarios can be expressed as simple operational templates. The central idea is that a genomic finding should be treated as a starting clue rather than a final verdict: A resistance interpretation should be assigned only when downstream evidence supports the implied mechanism, including immune activation state, antigen processing and presentation competence, spatial access of effector cells to tumor nests, suppressive myeloid or stromal programs, and microenvironmental metabolic constraints. Different genomic backgrounds may preferentially align with different dominant resistance axes, although these relationships should be interpreted as probabilistic rather than deterministic. In practice, EGFR‐mutant or ALK‐rearranged tumors may more often correspond to immune‐cold or immune‐excluded states, whereas KRAS‐mutant tumors with STK11 and/or KEAP1 coalterations may more often show suppression‐ or metabolic‐constraint–dominant phenotypes [63–65]. By contrast, TP53‐related inflammatory features appear to be context dependent and may coexist with substantial heterogeneity in clinical benefit, particularly across different comutation backgrounds [66]. This genotype‐informed but axis‐based interpretation may improve clinical usability by helping prioritize which downstream measurements are most informative in a given patient. In EGFR‐mutant or ALK‐rearranged disease, lower average benefit from checkpoint blockade has been observed clinically, but the underlying reasons are not uniform [67]. A practical workflow begins with genomic context and calibrated antigenicity proxies and then asks what the immune state actually looks like. In some tumors, low activation signals together with sparse effector‐cell infiltration on pathology support an immune‐cold explanation [68]. In others, immune activation may be detectable, yet immune cells remain spatially separated from tumor nests and stromal/exclusion signals are prominent, consistent with an inflamed‐but‐excluded pattern. Because different axes can dominate within the same driver‐defined context, the biomarker output should reflect the dominant mechanism rather than applying a single rule to all cases.

In KRAS‐mutant NSCLC with STK11 and/or KEAP1 coalterations, suppression and constraint have frequently appeared prominent in retrospective analyses, yet genotype alone is insufficient for a reliable interpretation [69]. A more transportable conclusion requires convergence across layers: attenuation of activation programs with enrichment of myeloid‐suppression modules, supportive spatial or pathology features consistent with exclusion or myeloid dominance, and evidence of metabolic constraints derived from metabolomics or robust proxy modules. When these signals align, resistance is more plausibly driven by a suppressive, metabolically restrictive microenvironment than by a simple lack of checkpoint target expression [70].

In TP53 coaltered contexts, inflammatory or interferon‐related signaling may be increased in some cohorts, but response remains variable. Practical discrimination can be improved by separating an inflamed and accessible state from an inflamed but adaptively resistant state. The latter is supported when high interferon activity coexists with high inhibitory programs and execution‐level evidence of suppression on proteomics or pathology, even in the presence of high PD‐L1. In contrast, preserved presentation competence and close spatial proximity of effector cells to tumor nests support an inflamed‐accessible state with a higher plausibility of benefit [71, 72].

High TMB with nonresponse illustrates why antigenicity alone is insufficient and why competence and spatial access must be interrogated. In this setting, nonresponse is often more consistent with impaired antigen processing/presentation (through genetic disruption or expression‐level silencing of key components), interferon signaling dysfunction, or exclusion‐ or suppression‐dominant architecture than with an absence of mutations. Accordingly, the most useful output is not that a TMB‐high tumor “should” respond, but a structured statement of which step in the recognition‐to‐killing chain appears to be limiting [73, 74].

For acquired resistance, single time‐point profiling can be misleading because the dominant mechanism may have emerged under treatment pressure. Acquired resistance is a dynamic process shaped by tumor–immune coevolution and may not be fully captured by baseline tissue alone. Accordingly, dynamic multiomics monitoring may add value beyond baseline prediction. Longitudinal strategies, including liquid biopsy and imaging follow‐up, may help track the trajectory of resistance evolution and identify emerging escape mechanisms [75]. A practical approach is to track changes along a few axes, such as declining presentation competence, newly attenuated interferon responsiveness, strengthening exclusion programs, expansion of suppressive myeloid states, or increasing metabolic constraints. Full multiomics reprofiling is rarely necessary; a focused longitudinal panel aimed at the leading hypothesized axis is often more feasible and can preserve interpretability while aligning with clinical reality [76, 77].

## 5. AI‐Based Model Construction for Multiomics Integration in NSCLC Immunotherapy

AI‐based multiomics integration should be formulated as a model‐building problem rather than a purely predictive exercise. The objective is to construct models that learn reproducible biological states from heterogeneous modalities, remain reliable when data are incomplete, and can be translated into compact, auditable outputs suitable for deployment. Accordingly, model construction can be organized around three design targets: learning stable representations across modalities, handling missing modalities with uncertainty‐aware inference, and distilling complex models into minimal evidence sets that preserve performance and interpretability [78].

Model development should begin with explicit attention to modality heterogeneity. Genomics, transcriptomics, epigenomics, proteomics, pathology, and metabolomics differ in scale, noise characteristics, batch structure, and sampling bias; accordingly, naive feature concatenation should be avoided, as it can amplify modality dominance and promote spurious shortcut learning driven by technical artifacts [79]. A more robust strategy is to learn modality‐specific embeddings using appropriate encoders and then perform alignment or fusion at a representation level. Fusion can be implemented through late‐fusion ensembles, gated or attention‐based fusion, or hierarchical architectures that separate tumor‐intrinsic signals from immune‐state programs [80]. Importantly, the learned representations should be constrained to remain interpretable by anchoring them to biologically meaningful immune‐state axes relevant to NSCLC immunotherapy and by mapping latent dimensions back to measurable modules or feature groups, rather than leaving them as opaque latent factors. Under current technological conditions, interpretability in multimodal fusion remains incomplete, but it is no longer methodologically absent. Relatively mature post hoc tools, including SHAP for complex models, are now available, and concept‐level and counterfactual analyses may further help assess whether model behavior is biologically and clinically plausible [81, 82]. However, these approaches should be regarded as supportive rather than definitive, since attention weights alone do not guarantee faithful explanation, and explanation stability may degrade under perturbation. Accordingly, “accountable AI” should be understood less as full transparency than as the capacity to generate auditable, robust, and clinically interpretable evidence trails for model outputs.

Because complete multiomics profiles are uncommon in real‐world NSCLC cohorts, missing‐modality handling should be built into both training and evaluation. Missingness is often systematic—driven by tissue availability, specimen adequacy, cost, and clinical urgency—so models trained only on complete cases typically overestimate performance and fail under deployment [83]. During model construction, missingness‐aware training can be implemented by modality dropout, mixture‐of‐experts architectures, or conditional fusion schemes that accept variable modality sets [84]. Outputs should include calibrated uncertainty estimates, so that low‐confidence predictions can be flagged for additional testing or conservative interpretation. Imputation may be used for supportive analyses, but it should not be treated as a substitute for uncertainty‐aware inference, and it should not hide the fact that key evidence is absent. A practical deployment strategy is graceful degradation: When only core modalities are available, the model outputs a baseline evidence set; when confirmatory modalities are available and concordant, confidence is increased and resistance phenotypes can be refined [85].

Model construction should also anticipate that translation rarely involves deploying a large multimodal network as is. Instead, complex models should be used to identify stable signal carriers and then distilled into compact, auditable measurement sets. Feature attribution, constrained modeling, and stability selection can be used to prioritize features that persist under platform shift and batch perturbation. Distillation can yield minimal evidence sets that combine calibrated antigenicity and mutational‐process features, competence checks, a limited number of activation/exclusion/suppression modules, and a few execution/constraint confirmations. Such outputs reduce operational burden, improve reproducibility, and facilitate external replication, while preserving the mechanistic interpretability expected of biomarker studies [86–88].

Evaluation should follow a stress‐testing paradigm aligned with deployment. In addition to reporting discrimination metrics, validation should explicitly test cross‐platform transfer, center shift, variation in tumor purity and cellular composition, and realistic missing‐modality patterns. Calibration performance and failure analysis should be reported, and operating boundaries should be stated clearly to indicate when the model is expected to be reliable and when uncertainty should dominate. Under this model‐building perspective, AI becomes a practical tool for constructing transportable, interpretable multiomics biomarkers for NSCLC immunotherapy resistance, rather than a means of maximizing performance in a single cohort [89].

In NSCLC immunotherapy, such panels may combine genomic context and PD‐L1/TMB–related information with targeted readouts of unresolved resistance axes, including antigen presentation, interferon competence, spatial exclusion, or metabolic suppression. However, practical implementation remains constrained by several persistent challenges, including limited tissue availability, assay heterogeneity, batch effects, cost, turnaround time, incomplete or systematically missing modalities, and the risk of overfitting in high‐dimensional settings with relatively modest sample sizes [90–92]. These issues argue against direct transfer of discovery‐scale workflows into routine care. A more feasible path is stepwise translation from broad discovery profiling to compact deployable panels, supported by modality‐aware normalization and harmonization, missingness‐aware model architectures, biologically constrained feature selection, cross‐platform and cross‐center stress‐testing, and prospective validation using lower burden assays compatible with routine pathology and molecular workflows.

## 6. Conclusions

Immunotherapy resistance in NSCLC is best represented as a multiaxis functional phenotype shaped by genetic variation, immune competence, adaptive resistance programs, spatial architecture, suppressive ecosystems, and metabolic constraints. Multiomics profiling enables genetic events to be interpreted as mechanistic hypotheses and translated into transportable biomarkers through coherent evidence chains spanning genomics, transcriptomics, epigenomics, proteomics, and metabolomics, complemented by spatial assays, digital pathology, and imaging‐derived surrogates.

AI can strengthen this process when it supports stable state representation, uncertainty‐aware inference under incomplete data, and disciplined compression into minimal evidence sets that remain auditable, standardizable, and independently verifiable [93].

Across development stages, coherence across layers, interpretability, and external transportability remain the criteria most directly linked to reproducible performance and credible mechanistic inference. Future progress is likely to depend less on increasing feature dimensionality and more on rigor in calibration, stress‐tested validation, longitudinal modeling of resistance trajectories, and construction of parsimonious measurement panels aligned with clinical workflows.

## Author Contributions

Yiqing Jiang conceived the topic and framework of the review, coordinated the writing process, critically revised the manuscript for important intellectual content, and served as the corresponding author. Na Wang and Qin Zeng conducted the literature search and screening, and drafted sections of the manuscript. Jun Liu and Xiaoqin Liu contributed to evidence synthesis, table/figure preparation, and refinement of key arguments. Guili Cao provided expert input, contributed to the interpretation of the literature, and critically revised the manuscript.

## Funding

No funding was received for this manuscript.

## Disclosure

All authors approved the final version of the manuscript and agreed to be accountable for all aspects of the work.

## Conflicts of Interest

The authors declare no conflicts of interest.

## Data Availability

Data sharing is not applicable to this article as no datasets were generated or analyzed during the current study.
